# Antidepressants and Gastric Cancer: A Nationwide Population-Based Nested Case-Control Study

**DOI:** 10.1371/journal.pone.0143668

**Published:** 2015-11-25

**Authors:** Yi-Hsuan Hsieh, Wei-Che Chiu, Chiao-Fan Lin, Hsiang-Lin Chan, Hsin-Yi Liang, Yena Lee, Roger S. McIntyre, Vincent Chin-Hung Chen

**Affiliations:** 1 Department of Child and Adolescent Psychiatry, Linkou Chang Gung Memorial Hospital, Taoyuan, Taiwan; 2 Department of Psychiatry, Cathay General Hospital, Taipei 10630, Taiwan; 3 School of Medicine, Fu Jen Catholic University, Taipei 24205, Taiwan; 4 Department of Psychiatry, University of Toronto, Mood Disorders Psychopharmacology Unit, University Health Network, Toronto, Canada; 5 Department of Psychiatry, Chiayi Chang Gung Memorial Hospital, Chiayi, Taiwan; Chiba University Center for Forensic Mental Health, JAPAN

## Abstract

**Background:**

To our knowledge, no epidemiological study has reported on whether an association between antidepressant exposure and gastric cancer exists. Herein, we aim to investigate the possible association between antidepressant exposure and gastric cancer incidence.

**Methods:**

Using a nested case-control design, we identified 26289 cases with gastric cancer and 127984 controls from Taiwan’s National Health Insurance Research Database (NHIRD). The data were analyzed using a conditional logistic regression model adjusting for possible confounding variables.

**Results:**

We found antidepressant use did not increase the risk of gastric cancer. The lack of an association between antidepressant prescription and elevated gastric cancer incidence was apparent for across selective serotonin-reuptake inhibitors (SSRIs), tricyclic agents (TCAs), serotonin-norepinephrine reuptake inhibitors (SNRIs), reversible inhibitors of monoamine oxidase A (RIMA), trazodone, mirtazapine and bupropion. There were slightly decreased gastric cancer risks of SSRIs use (≧28 DDD group, adjusted OR = 0.87; 95% CI = 0.78–0.96). Sensitive analysis showed SSRIs, TCAs, and SNRIs did not increase gastric cancer risks significantly even in the group with peptic ulcer history.

**Conclusions:**

An association between antidepressant exposure and gastric cancer was not apparent in this analysis.

## Introduction

Gastric cancer is the third and fifth most common cause of mortality attributable to cancer in men and women, respectively, resulting in 738000 deaths per year worldwide[1]. It has been identified that peptic ulcer disease is a risk factor for incident gastric cancer. A large cohort study in Sweden reported that patients with gastric ulcer have an approximate 10-fold greater risk of gastric cancer in the first 2 years after diagnosis of peptic ulcer disease compared to those without peptic ulcer disease[2]. A separate cohort study in Taiwan reported that the relative risk for gastric cancer was 1.49–1.82 in individuals with gastric ulcer compared to those without[3].

In addition to studies reporting the relationship between gastric ulcer disease and gastric cancer, other studies have reported on a possible association between antidepressant prescription and gastric cancer incidence. Preclinical evidence indicates that exposure to antidepressants [e.g. tricyclic agents (TCAs), selective serotonin-reuptake inhibitors (SSRIs) and serotonin-norepinephrine reuptake inhibitors (SNRIs)] is associated with gastro-protective effects[4–8]. Specifically, augmentation of the innate antioxidant systems, as well as reduction in gastric secretion has been proposed as mechanisms mediating the gastroprotective effect of antidepressants[8].

Very different results were published by Takeuchi et al, who reported that paroxetine aggravated indomethacin-induced antral damage in rats, with a hypothesis that the effect was mediated via the activation of 5HT3 receptors[9]. Results from original reports and review articles have consistently identified an association between SSRI exposure and upper gastrointestinal bleeding; a risk that was exacerbated by concomitant exposure to anti-inflammatory agents, anti-coagulants and antiplatelet agents[10]. In a population-based cohort study in Denmark, combined use of an SSRI and nonsteroidal anti-inflammatory drugs or low-dose aspirin increased the risk to 12.2 (95% CI = 7.1–19.5) and 5.2 (95% CI = 3.2–8.0)[11]. Results from a recent meta-analysis further support the foregoing result indicating that there is an increase in risk both in the case–control studies (OR = 1.66, 95% CI = 1.44–1.92) and cohort studies (OR = 1.68, 95% CI = 1.13–2.50), and the risk of upper GI bleeding was further increased with the use of both SSRIs and NSAID medications (OR = 4.25, 95% CI = 2.82–6.42)[12]. Recently a case-crossover study in Taiwan reported that short-term SSRIs exposure (i.e. 7–28 days) was significantly associated with upper gastrointestinal bleeding[13].

A derivative of the foregoing collection of observations is the testable hypothesis that antidepressant exposure may be associated with malignant changes. A large prospective cohort study in Denmark reveled no overall increased cancer risk among antidepressants users, except for a possible effect of tricyclic antidepressants and tetracyclic antidepressants on non-Hodgkin’s lymphoma[14]. A population-based case-control study reported that use of TCAs did not increase the risk for incident gastric cardia adenocarcinoma. A limitation, however, of this analysis was a relatively small sample size[15]. A record linkage from Finland utilizing nationwide databases did not identify any association between antidepressant exposure and incident gastric carcinoma[16]. Moreover, experimental data reported that mirtazapine prevented adenocarcinoma induction by N-methyl-N-nitro-N-nitrosoguanidine (MNNG) in rats to a greater extent than cisplatin[17].

The relatively high prevalence of gastric cancer in some countries/regions as well as the preliminary pre-clinical and epidemiologically associated reports attempting to determine whether antidepressants have risk-enhancing (or lowering) effects on gastric cancer provided the impetus for the analysis herein. We sought to determine whether any association existed between antidepressant exposure and gastric cancer in a large cross-national cohort of adults registered in the Taiwan’s National Health Insurance Research Database (NHIRD). We also took into consideration multiple confounding factors that may have inadvertently affected outcomes of interest.

## Materials and Methods

### Source population

The National Health Insurance program in Taiwan has been in operation since 1995. It covered over 23 million residents by 2010, which represented approximately 99% of Taiwan’s population. The nationwide population-based nested case-control study NHIRD contains comprehensive health information such as patients’ socio-demographic data, diagnostic codes, medical procedures, and prescriptions is recorded in the NHIRD. The population of this study was derived from the NHIRD between January 1, 1997 and December 31, 2008. The study data set includes no patient identification information thereby rendering it unnecessary to obtain the approval of an institution review board.

### Cases of gastric cancer and controls

In Taiwan, patients with definite cancer diagnosis would register with the Catastrophic Illness Registry and apply for a catastrophic illness certificate.

We defined cases of gastric cancer by ICD-9-CM codes (151.xx) from NHIRD, and confirmed the diagnosis by linkage to the Catastrophic Illness Registry Dataset. Cases were defined as newly diagnosed with gastric cancer between January 1, 1999 and December 31, 2008. Individuals with gastric cancer who had any previous cancer diagnoses in the dataset between January 1, 1997 and December 31, 1998 were excluded. The date of the first gastric cancer claim was defined as the index date.

**Fig 1:**
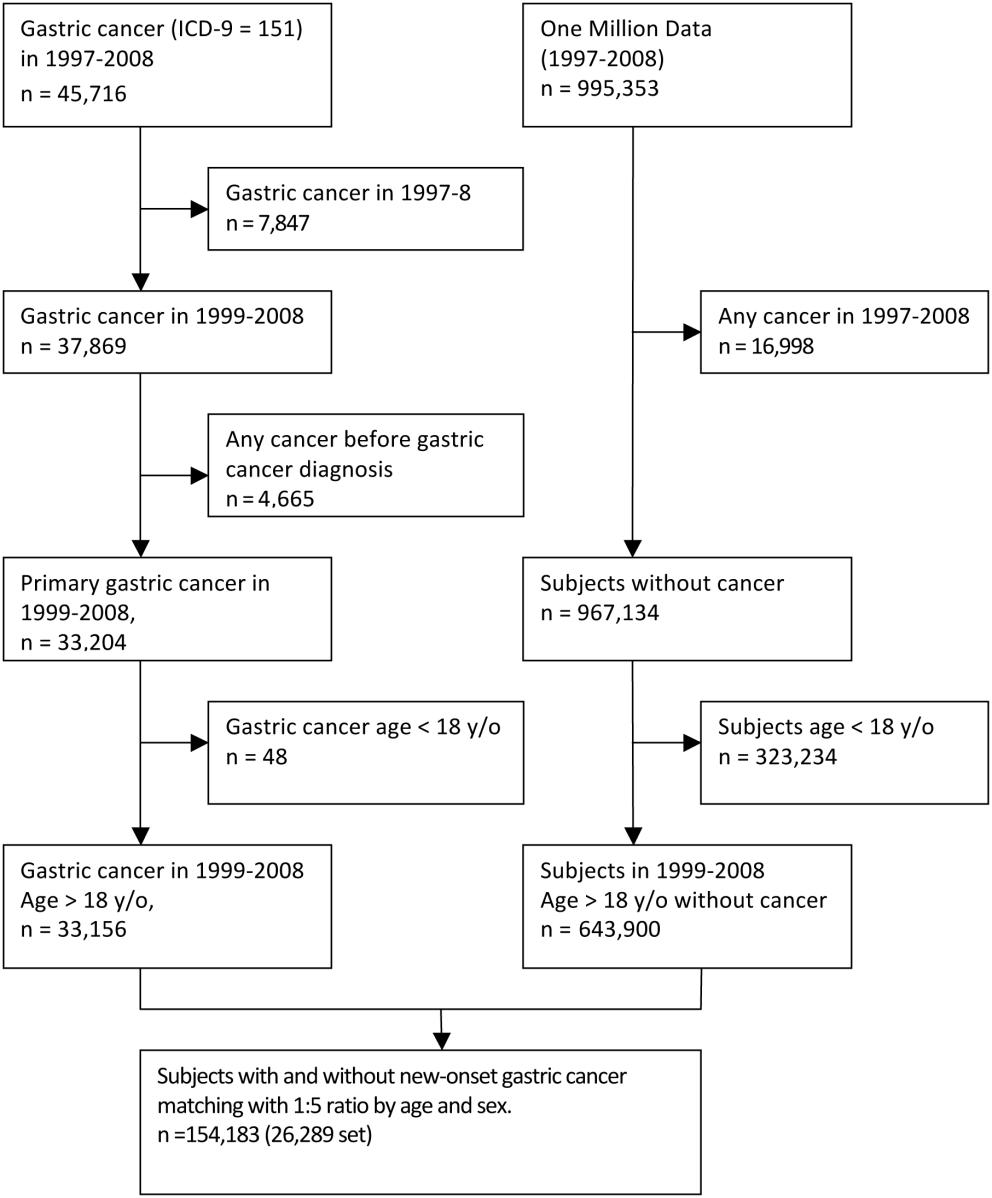
Flow chart of selection subjects.

We used an incidence density sampling method for each gastric cancer case and randomly selected five controls without gastric cancer diagnosis at the index date of gastric cancer cases[18]. The dataset for the control population of 1 million samples was randomly culled from the entire NHI population, and members who were free of a cancer diagnosis were selected for the control population. The controls were individually matched with gastric cancer cases by year of birth and sex (Fig 1).

### Antidepressants’ exposure

Antidepressants (N06A) were identified based on the Anatomical Therapeutic Chemical classification system. The data of exposure to the antidepressant and related drug information (i.e., the cumulative dose) was retrieved from the NHIRD. In our study, antidepressants were classified into SSRIs (e.g. fluoxetine, fluvoxamine, paroxetine, citalopram, escitalopram, and sertraline), SNRIs (e.g. duloxetine and venlafaxine), NaSSA (e.g. mirtazapine), TCAs (e.g. amitriptyline, clomipramine, imipramine, dothiepin, doxepin, maprotilinem, and melitracen), SARI (e.g. trazodone), RIMA (e.g. moclobemide), and NDRI (e.g. bupropion).

In order to avoid protopathic bias, all drug exposures in the duration of one year immediately prior to the index date were excluded[19]. Each patient’s exposure to an antidepressant was determined using the cumulative dose of antidepressants, which is quantified by a defined daily dose [DDD][20]. The cumulative doses were designed to four graded exposure dose levels: (1) ≧28 DDD, (2) ≧84 DDD, (3) ≧168 DDD, (4) ≧336 DDD.

### Demographic data and potential confounding factors

We extracted the patients’ socio-demographic variables including age, gender, income range and urbanization from the NHIRD. Comorbid mental and physical diseases, procedures, and other medication use were regarded as confounding factors and assessed using outpatient and inpatient claims records before the index date. We listed depressive disorders and anxiety disorders as comorbid mental disorders, and adjusted for type 2 diabetes mellitus (T2DM), hypertension, hypercholesterolemia, heart failure, coronary heart disease (CHD), peptic ulcer, asthma, chronic obstructive pulmonary disease (COPD), chronic kidney disease (CKD), liver cirrhosis, and cerebral vascular accident (CVA). The administration of potentially confounding drugs, including proton pump inhibitors (PPIs), triple therapy for H. pylori eradication (combination of PPIs, amoxicillin and clarithromycin), nonsteroidal anti-inflammatory drugs (NSAIDs), aspirin and statins which prescribed before the index date was also confirmed. Furthermore, there was a positive association between heavy alcohol drinking and gastric cancer risk[21], but alcohol drinking status was not documented in the NHIRD; instead, alcohol-related disorders were examined.

### Statistical analysis

Descriptive statistics of gastric cancer cases and controls were reported in terms of their demographic characteristics, comorbid disorders, medical and alcohol use.

To find out if there is any association between antidepressants use and gastric cancer risk, we used the PHREG procedure of SAS (version 9.2) to carry out conditional logistic regression models. The different classes of antidepressants use and four cumulative dose levels were assessed separately. Corrected odds ratios are evaluated with adjustment for demographic data and confounding factors.

A p-value less than 0.05 or a 95% confidence interval (CI) was used to indicate statistical significance. All statistical analyses were performed using SAS version 9.2 (SAS Institute, Cary, NC).

## Results

Cases and controls were matched on the basis of sex and age; a total of 26289 cases with a diagnosis of gastric cancer and 127984 controls were identified between January 1, 1997 and December 31, 2008. Descriptive demographic data, e.g., age, sex, income and urbanization, are shown in Table 1. The mean age at the first-time gastric cancer diagnosis was 58.2 ±13.6 years old; males had higher risk of gastric cancer (male: 57.13%; female: 42.87% in cases group).

**Table 1:** Demographic Data of Cases and Controls.

		Cases (n = 26289)	Controls (n = 127984)	p value
**Sex**	M	15020 (57.13%)	72868 (56.94%)	0.55
	F	11269 (42.87%)	55116 (43.06%)	
**Age**	≦40	2880 (10.96%)	14068 (10.99%)	0.62
	41–50	4764 (18.12%)	23469 (18.34%)	
	51–60	5553 (21.12%)	27307 (21.34%)	
	61–70	7576 (28.82%)	36852 (28.79%)	
	71–80	4736 (18.02%)	22652 (17.70%)	
	≧80	780 (2.97%)	3636 (2.84%)	
**Income(NTD** ^a^)	0	5169 (19.66%)	24443 (19.10%)	<0.0001
	1–25000	4171 (15.87%)	20908 (16.34%)	
	25001–40000	12292 (46.76%)	57852 (45.20%)	
	≧40001	4657 (17.71%)	24781 (19.36%)	
**Urbanization** ^b^	Very high	7539 (28.68%)	35903 (28.05%)	<0.0001
	High	12040 (45.80%)	57518 (44.94%)	
	Moderate	4530 (17.23%)	23050 (18.01%)	
	Low	2180 (8.29%)	11513 (9.00%)	

^a^ 1US $ = 32.3 New Taiwan Dollars (NTD) in year 2008

^b^ Quartiles by human development index

We provide descriptive information as it relates to comorbid mental and physical disorders, and medication use (except antidepressants) in Table 2. The incidence rates of T2DM, hypertension, heart failure, peptic ulcer, asthma, COPD, alcohol-related disease, CKD, liver cirrhosis were all significantly higher in the cancer group than in the control group. In addition, individuals with gastric cancer were also significantly more likely to have received more PPIs, triple therapy (combination of clarithromycin, amoxicillin, and PPIs) and NSAIDs.

**Table 2:** Medical Diseases and Drugs Used with Cases and Controls.

	Cases (n = 26289)	Controls (n = 127984)	p value
**Mental/physical disorders**			
**Depressive disorder** ^a^	767 (2.92%)	4110 (3.21%)	0.013
**Anxiety disorder**	3108 (11.82%)	15583 (12.18%)	0.11
**Type 2 DM**	4096 (15.58%)	17058 (13.33%)	<0.0001
**Hypertension**	7837 (29.81%)	32409 (25.32%)	<0.0001
**Hypercholesterolemia**	2932 (11.15%)	14897 (11.64%)	0.025
**Heart failure**	999 (3.80%)	4115 (3.22%)	<0.0001
**CHD**	3731 (14.19%)	17237 (13.47%)	0.0018
**Peptic ulcer**	6183 (23.52%)	22458 (17.55%)	<0.0001
**Asthma**	2940 (7.54%)	9654 (5.08%)	<0.0001
**COPD**	3982 (15.15%)	17529 (13.70%)	<0.0001
**Alcohol disease**	145 (0.55%)	370 (0.29%)	<0.0001
**CKD**	574 (2.18%)	1679 (1.31%)	<0.0001
**Liver cirrhosis**	2782 (10.58%)	11438 (8.94%)	<0.0001
**CVA**	2436 (9.27%)	11970(9.35%)	0.66
**Medications**			
**Triple therapy** ^b^	983(3.74%)	3667 (2.87%)	<0.0001
**PPIs**	2436(9.27%)	8272 (6.46%)	<0.0001
**Aspirin**	4578(17.41%)	22411 (17.51%)	0.71
**NSAIDs** ^c^	14933(56.80%)	74218 (57.99%)	0.0004
**Statins**	1692(6.44%)	8644 (6.75%)	0.061

^a^ Depressive disorder (ICD-9: 296.2, 296.3, 300.4, 311).

^b^ Combination of PPIs, amoxicillin and clarithromycin

^c^ NonSteroidal anti-inflammatory drugs

The main findings are shown in Table 3, with the results after adjusting with urbanization, income, hypertension, diabetes, hypercholesterolemia, chronic kidney disease, peptic ulcer and depressive disorder. Small case numbers had ever been prescribed mirtazapine and bupropion at least one year before the index date (<0.1%). There was no significant difference between cancer cases and controls regardless of cumulative dose across SNRIs, RIMA, mirtazapine and bupropion treatments. There were slightly decreased gastric cancer risks of SSRIs use (≧28 DDD, adjusted OR = 0.87; 95% CI = 0.78–0.96; ≧84 DDD, adjusted OR = 0.86; 95% CI = 0.75–0.97), TCAs use (≧28 DDD, adjusted OR = 0.89; 95% CI = 0.82–0.96) and trazodone use (≧168 DDD, adjusted OR = 0.64; 95% CI = 0.45–0.90).

**Table 3:** Associations of Antidepressants Use and Gastric Cancer Risk.

Antidepressants	Cases (n = 49342)		Controls (n = 240985)		OR	
					Crude (95%CI^a^)	Adjusted^b^ (95% CI)
	N	%	N	%		
**SSRI** ^c^						
**≧28DDD**	598	2.27	3261	2.55	0.88 (0.80–0.96)	0.87 (0.78–0.96)
**≧84DDD**	343	1.30	1936	1.51	0.84 (0.75–0.95)	0.86 (0.75–0.97)
**≧168DDD**	240	0.91	1317	1.03	0.87 (0.76–1.00)	0.90 (0.78–1.05)
**≧336DDD**	139	0.53	817	0.64	0.81 (0.68–0.97)	0.86 (0.71–1.04)
**SNRI** ^d^						
**≧28DDD**	47	0.18	268	0.21	0.83 (0.61–1.13)	0.87 (0.63–1.20)
**≧84DDD**	36	0.14	182	0.14	0.94 (0.66–1.35)	0.99 (0.69–1.43)
**≧168DDD**	19	0.07	125	0.10	0.73 (0.45–1.18)	0.77 (0.47–1.26)
**≧336DDD**	13	0.05	75	0.06	0.83 (0.46–1.51)	0.90 (0.50–1.64)
**TCA** ^e^						
**≧28DDD**	882	3.36	4358	3.41	0.96 (0.89–1.04)	0.89 (0.82–0.96)
**≧84DDD**	432	1.64	1958	1.53	1.06 (0.95–1.17)	0.99 (0.89–1.10)
**≧168DDD**	214	0.81	1010	0.79	1.01 (0.87–1.18)	0.96 (0.83–1.12)
**≧336DDD**	93	0.35	415	0.32	1.08 (0.86–1.35)	1.05 (0.84–1.33)
**RIMA** ^f^						
**≧28DDD**	161	0.61	781	0.61	1.00 (0.84–1.18)	1.00 (0.84–1.19)
**≧84DDD**	82	0.31	421	0.33	0.94 (0.74–1.19)	0.96 (0.75–1.22)
**≧168DDD**	47	0.18	252	0.20	0.90 (0.66–1.22)	0.94 (0.69–1.30)
**≧336DDD**	28	0.11	132	0.10	1.02 (0.68–1.53)	1.10 (0.73–1.67)
**Mirtazapine**						
**≧28DDD**	19	0.07	91	0.07	0.99 (0.60–1.62)	1.05 (0.64–1.74)
**≧84DDD**	9	0.03	49	0.04	0.88 (0.43–1.79)	0.94 (0.46–1.92)
**≧168DDD**	5	0.02	26	0.02	0.91 (0.35–2.36)	1.00 (0.38–2.63)
**≧336DDD**	2	0.01	10	0.01	0.94 (0.21–4.31)	1.06 (0.23–4.87)
**Trazodone**						
**≧28DDD**	305	1.16	1449	1.13	1.00 (0.89–1.14)	1.00 (0.87–1.13)
**≧84DDD**	117	0.45	630	0.49	0.88 (0.72–1.06)	0.90 (0.73–1.10)
**≧168DDD**	38	0.14	290	0.23	0.62 (0.44–0.87)	0.64 (0.45–0.90)
**≧336DDD**	13	0.05	88	0.07	0.69 (0.39–1.24)	0.74 (0.41–1.33)
**Bupropion**						
**≧28DDD**	9	0.03	37	0.03	1.16 (0.56–2.40)	1.19 (0.57–2.48)
**≧84DDD**	2	0.01	23	0.02	0.40 (0.09–1.69)	0.41 (0.10–1.75)
**≧168DDD**	1	0.00	12	0.01	0.39 (0.05–3.03)	0.41 (0.05–3.18)
**≧336DDD**	0	0.00	5	0.00	-	-

^a^ confidence interval

^b^ Adjusting with urbanization, income, hypertension, diabetes, hypercholesterolemia, chronic kidney disease, peptic ulcer and depressive disorder.

^c^ SSRI: selective serotonin re-uptake inhibitors

^d^ SNRI: Serotonin–norepinephrine reuptake inhibitors

^e^ TCA: tricyclic antidepressants

^f^ RIMA: Reversible inhibitors of monoamine oxidase A

To further explore the relationships between antidepressants use, peptic ulcer and gastric cancer, we performed sensitive analysis of antidepressants on gastric cancer risk in the patients with or without peptic ulcer (Table 4). Three classes of antidepressant (SSRIs, SNRIs, and TCAs) were selected because of recent antidepressants using trends. We did not find any association/interaction between antidepressant prescription, peptic ulcer disease and gastric cancer. Only SNRIs use was slightly associated with increased gastric cancer risk in patients who had history of peptic ulcer (≧28 DDD, adjusted OR = 1.26; 95% CI = 0.73–2.16; ≧84 DDD, adjusted OR = 1.36; 95% CI = 0.73–2.53; ≧168 DDD, adjusted OR = 1.34; 95% CI = 0.62–2.93; ≧336 DDD, adjusted OR = 2.00; 95% CI = 0.77–5.20); the foregoing association remained insignificant across all four cumulative dose levels.

**Table 4:** Subgroup Analysis Antidepressants on Gastric Cancer Risk in the Patients with or without Peptic Ulcer.

Antidepressants	Adjusted^b^ OR (95%CI^a^)	
	History of peptic ulcer (n = 28641)	Without peptic ulcer (n = 125632)
**SSRI** ^c^		
**≧28DDD**	0.89 (0.72–1.09)	0.87 (0.76–1.00)
**≧84DDD**	0.89 (0.69–1.14)	0.86 (0.71–1.04)
**≧168DDD**	0.95 (0.71–1.28)	0.87 (0.70–1.08)
**≧336DDD**	0.87 (0.61–1.24)	0.78 (0.59–1.03)
**SNRI** ^d^		
**≧28DDD**	1.26 (0.73–2.16)	0.65 (0.36–1.15)
**≧84DDD**	1.36 (0.73–2.53)	0.75 (0.37–1.55)
**≧168DDD**	1.34 (0.62–2.93)	0.70 (0.29–1.68)
**≧336DDD**	2.00 (0.77–5.20)	0.82 (0.28–2.43)
**TCA** ^e^		
**≧28DDD**	0.91 (0.78–1.06)	0.92 (0.83–1.03)
**≧84DDD**	0.98 (0.79–1.22)	1.04 (0.89–1.21)
**≧168DDD**	0.94 (0.70–1.26)	0.99 (0.80–1.23)
**≧336DDD**	1.35 (0.89–2.07)	0.92 (0.66–1.28)

^a^ confidence interval

^b^ Adjusting with urbanization, income, hypertension, diabetes, hypercholesterolemia, chronic kidney disease, depressive disorder, aspirin, nonsteroidal anti-inflammatory drugs and triple therapy

^c^ SSRI: selective serotonin re-uptake inhibitors

^d^ SNRI: Serotonin–norepinephrine reuptake inhibitors

^e^ TCA: tricyclic antidepressants

## Discussion

To our knowledge, this is the first study to evaluate whether an association exists between antidepressant prescription and gastric cancer in a large cross-national database adjusting for multiple confounding factors. Our primary finding is that we were unable to identify any association between antidepressant prescriptions and increased (or decreased) gastric cancer risk. The lack of an association between antidepressant exposure and gastric cancer risk remained after adjusting for different antidepressant cumulative dosing.

The overall lack of an association between antidepressant exposure and gastric cancer is directionally consistent with findings from two previous reports [15, 16]. For example, Vaughan *et al*. reported that TCAs prescription did not increase gastric cardia adenocarcinoma risk in a population-based case control study with 260 cases (adjusted OR = 0.3; 95% CI = 0.1–1.2)[15]. A limitation, however, of the foregoing report is that only 3 subjects within the cancer group were ever prescribed a TCA, raising the strong possibility of an underpowered study. Our larger study (i.e. 882 people used TCAs in 49342 cancer patients) reported similar findings.

In Finland, Haukka *et al*. conducted a record linkage study involving 594 gastric cancer cases[16]. The RR of gastric cancer in the highest exposure category to SSRIs (more than 1,460 days) was 1.16 (95% CI = 0.49–2.76). However, compared to their study, our large sample size of individuals with gastric cancer (26289 cases) enabled us to further subgroup individuals prescribed non-SSRIs into: SNRIs, TCAs, RIMA, SARI, NaSSA, and NDRI. Notwithstanding the differences between racial, ethnic and cultural groups, the results from our study, which was largely a Han Chinese population, cohere with results with European samples.

We observed that in addition to having a non-elevation of cancer risk, there was a slight decrease in overall cancer risk associated with SSRI prescription (≧28 DDD group and ≧84 DDD group), TCAs prescription (≧28 DDD group) and trazodone prescription (≧168 DDD group). The foregoing results should, however, be interpreted cautiously as the sample sizes were relatively small (i.e., trazodone). Moreover, we did not find a “dose-response curve” insofar as greater exposure, as proxied by greater antidepressant cumulative dosing, was not associated with decreased risk. Available pre-clinical studies provide evidence directionally consistent with the hypothesis that SSRIs, SNRIs, and TCAs may have gastro-protective effects[4–8]. The foregoing gastro-protective effects, however, need to be taken in a clinical context as replicative evidence also indicates that SSRIs exposure is associated with increased upper gastrointestinal bleeding [10, 12]. Moreover, the association between SSRI prescription and upper GI bleeding, which differentially affects older populations receiving concomitant medications known to affect chemostatis was absorbed after relatively short term exposure to antidepressants (i.e., 7 days)[13].

In our current analysis we further subgrouped the sample according to peptic ulcer history. We were unable to find any association between SSRIs, TCAs, SNRIs, and a history of peptic-ulcer disease and gastric cancer.

## Limitations and Strengths

There are several methodological limitations that affect inferences and interpretations that may be drawn from our data. For example, several confounding factors such as lifestyle, diet, occupation and smoking were not acquired in our database. Second, using pharmacy records representing dispensing data might overestimate antidepressants cumulative doses because drug adherence could not be determined. Third, we separately analyzed the gastric cancer risks of distinct antidepressants classes, and the relatively small percentage of SNRIs, NaSSA and NDRI use may weaken the power of statistics in these three classes.

Strengths of our data are that our results are extracted from a population-based nested case-control design and socio-demographic data, diagnostic codes, medical procedures and medical prescription data were extracted from NHIRD. The possibility of recall and selection bias was reduced via this method. Notwithstanding the large sample size along with the requirement that medical diagnosis established by a physician, the use of the ICD-9-CM mitigates recall biases. Another strength of our study was that we were able to look at the temporal relationship between antidepressant exposure and gastric cancer, providing a suggestion, but certainly not confirmation, of causality if it existed.

## Conclusion

In summary, our study demonstrates that there was no association between risk of gastric cancer and the prescription of antidepressant drugs. Our results are highly clinically relevant insofar that in some regions of the world, antidepressant prescription is increasing with more adults receiving antidepressants for longer periods of time. We believe that the absence of an association between antidepressant prescription and gastric cancer provides confidence that very serious gastric pathology would not be expected with longer-term antidepressant exposure.
